# Supplementary material for: Classical non-homologous end-joining pathway utilizes nascent RNA for error-free double-strand break repair of transcribed genes
Source: Nat Commun. 2016 Oct 5;7:13049. doi: 10.1038/ncomms13049 (PMC5059474; doi:10.1038/ncomms13049)
Supplement: Supplementary Information — Supplementary Figures 1-8 and Supplementary Tables 1-2 [file ncomms13049-s1.pdf]

Supplementary Figure 1

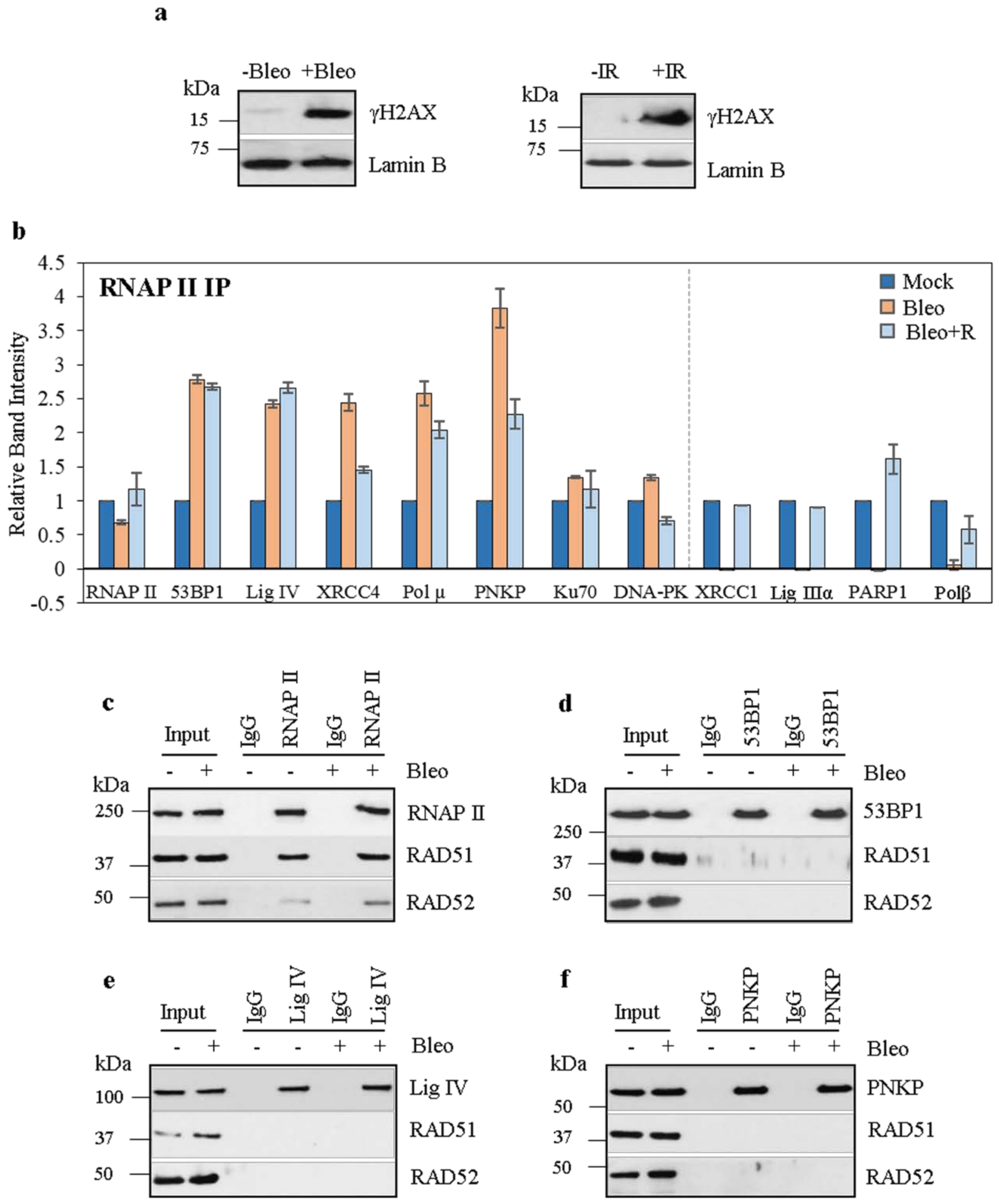

Supplementary Figure 1: Characterization of DSB repair complexes by co-IP analysis

(a) Western blot analysis to estimate the level of γH2AX (phosphorylated form) in NE from mock/Bleo-treated (left panel) and mock/IR-exposed (right panel) HEK293 cells. Lamin B was used as a loading control in each case. **(b)** The intensities of the IP'd protein bands were estimated from three independent experiments from separate batches of cells for Figure 1a. Error bars represent ± SD of the mean. NE (benzonase-treated) from HEK293 cells [either mock (-) or Bleo (+) treated] were IP'd with **(c)** anti-RNAP II (pSer2, H5 Ab); **(d)** anti-53BP1; **(e)** anti-Lig IV; **(f)** anti-PNKP antibodies (Abs, even lanes) or control IgG (odd lanes) and tested for the presence of associated proteins with specific Abs as indicated to the right of each row. Each experiment was repeated at least three times with separate batches of cells, and one representative figure is shown in each case.

Supplementary Figure 2

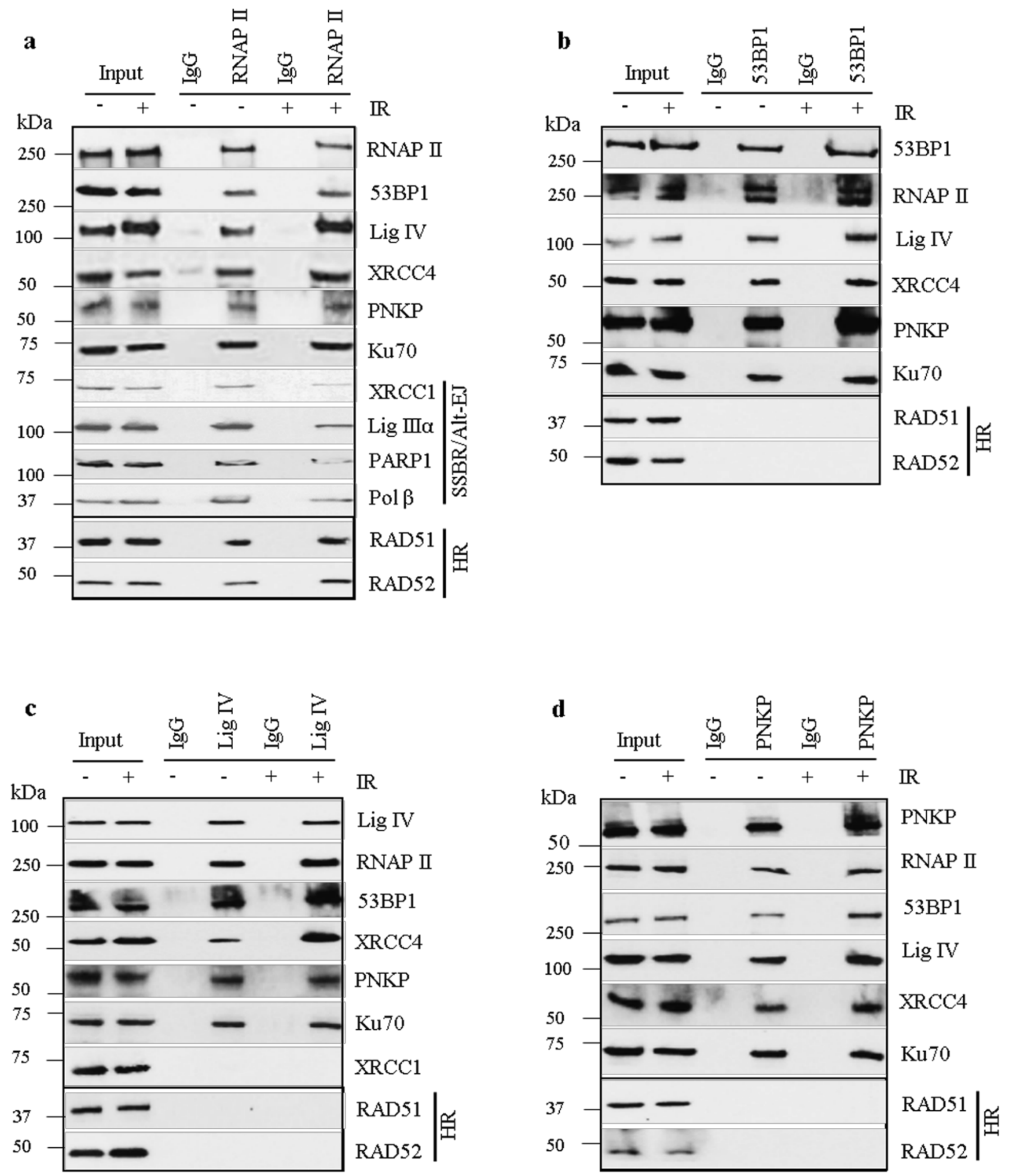

Supplementary Figure 2: Partial characterization of ionizing radiation-induced DSB repair complexes by co-IP analysis

NE (benzonase-treated) from HEK293 cells [either mock (-) or IR (+) exposed] were IP'd with **(a)** anti-RNAP II (pSer2, H5 Ab); **(b)** anti-53BP1; **(c)** anti-Lig IV; **(d)** anti-PNKP antibodies (Abs, even lanes) or control IgG (odd lanes) and tested for the presence of associated proteins with specific Abs as indicated to the right of each row. Each experiment was repeated at least three times with separate batches of cells with independent IR exposure, and one representative figure is shown in each case.

Supplementary Figure 3

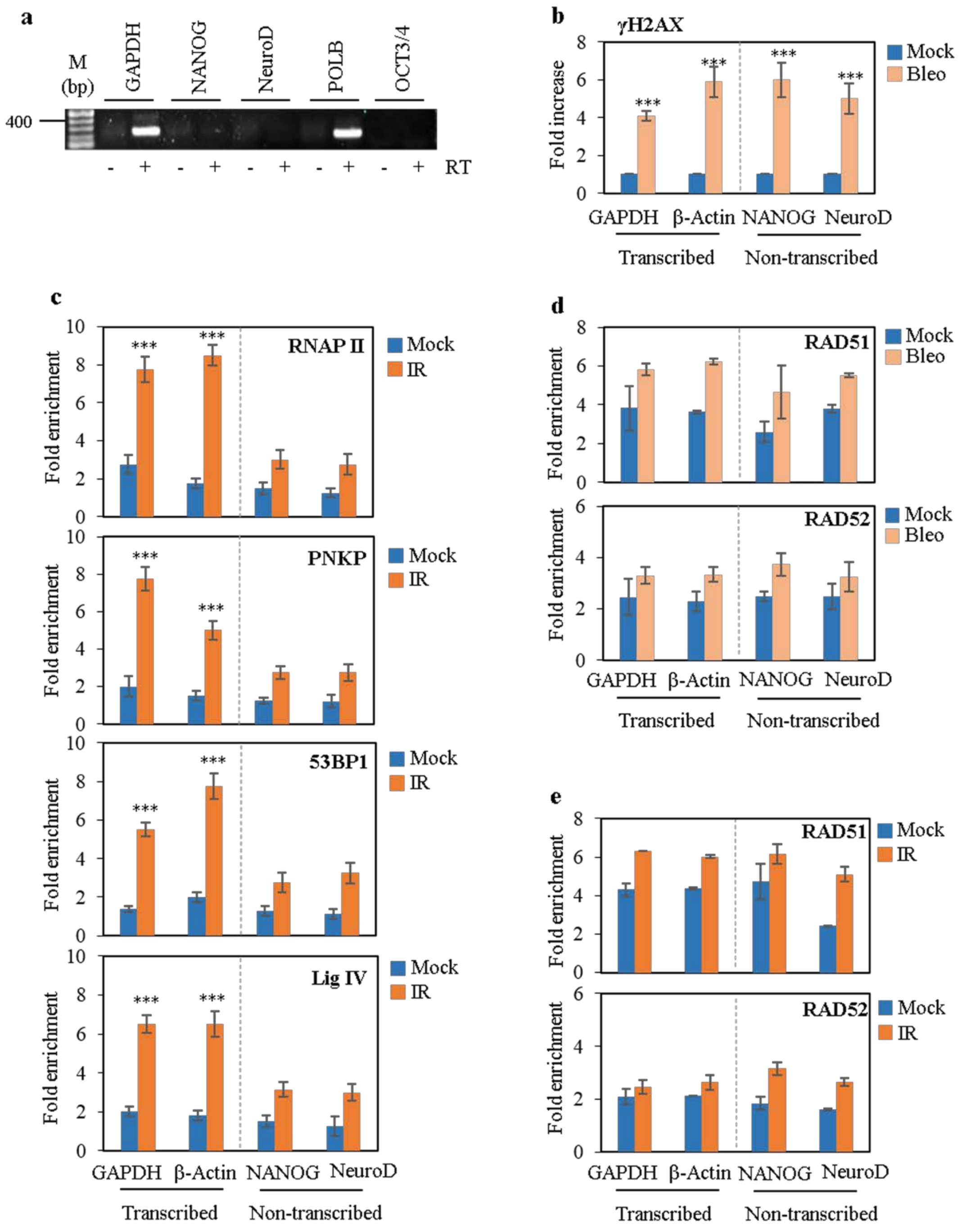

Supplementary Figure 3: Quantitative ChIP assay to determine the association of C-NHEJ/ HR proteins with transcribed vs non-transcribed genes

(a) Gene expression profile of selected genes used in the studies presented in Figs. 2 and 3.

The shown PCR fragments were obtained from cDNA generated by RT reaction with total RNA of HEK293 cells. As a control for DNA contamination, no RT enzyme was added in all "-RT" samples. All oligos used for amplification of the genes are listed in Supplementary Table 1.

(b) HEK293 cells were mock- or Bleo-treated and ChIP was performed with anti-Histone H2A.XS 139ph (ser phos 139 residue) Ab. Binding to the exonic regions of transcribed (GAPDH and ß-Actin) vs non-transcribed (NANOG and NeuroD) genes was quantified by qPCR from IP'd DNA. The data are represented as fold increase in normalized percent input (over IgG) with mock samples considered as unity. Error bars represent ±SD of the mean (n=3). ***=P<0.005 represents statistical significance between mock- and Bleo-treated samples. **(c)** HEK293 cells were mock- or IR-exposed and ChIP was performed with specific Abs as indicated. Binding to the exonic regions of transcribed (GAPDH and ß-Actin) vs non-transcribed (NANOG and NeuroD) genes was quantified by qPCR from IP'd DNA. The data are represented as fold enrichment of percent input of the sample over IgG. Error bars represent ±SD of the mean (n=3). ***=P <0.005 represents statistical significance within a treatment group between a particular transcribed gene with both non-transcribed genes (NANOG and NeuroD).

ChIP assays were performed with NE from mock/Bleo-treated **(d)** and mock/IR-exposed **(e)** HEK293 cells using anti-RAD51 or -RAD52 Abs and analyzed by qPCRs using specific primers for transcribed and non-transcribed genes. Error bars represent ±SD of the mean calculated from three independent experiments.

Supplementary Figure 4

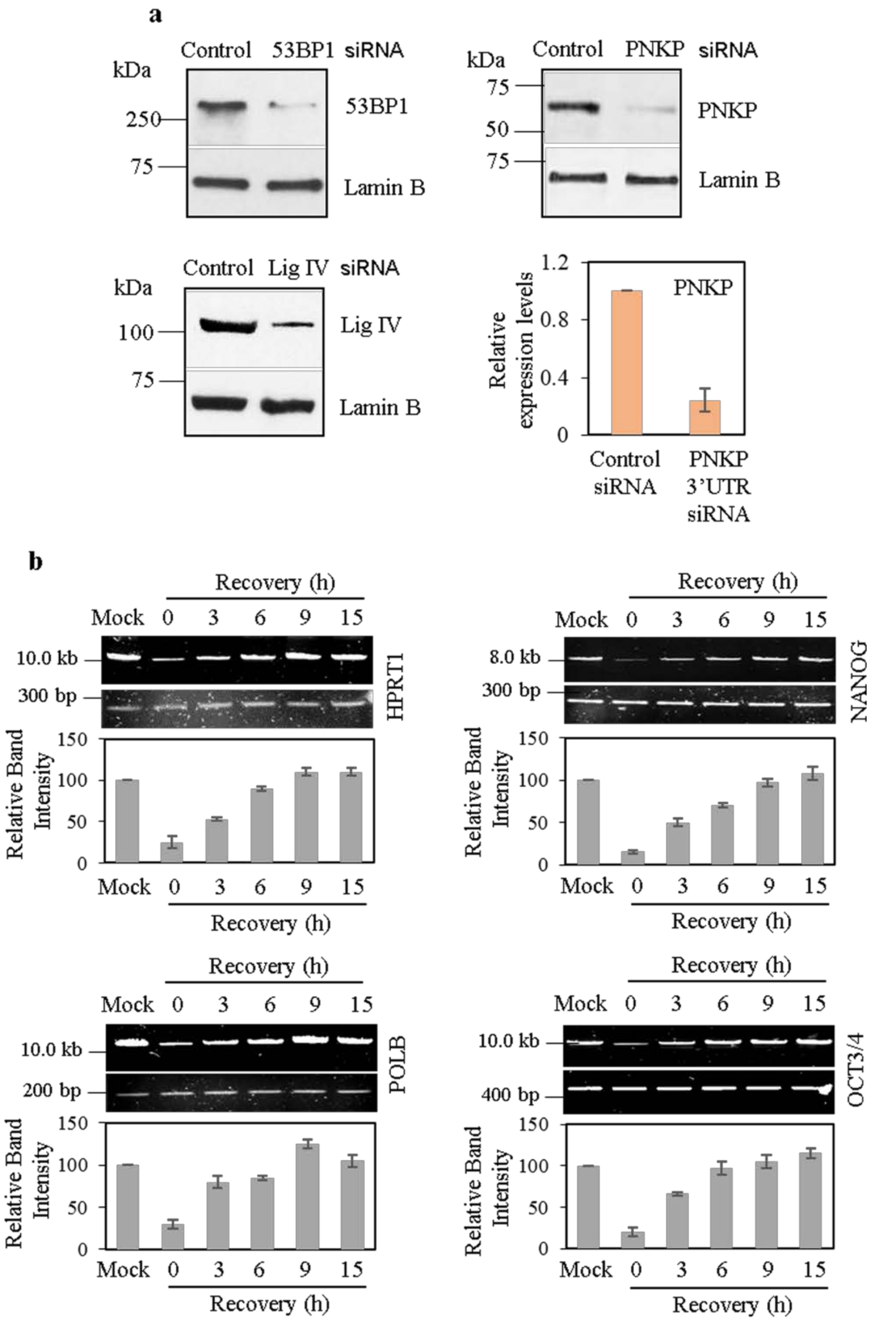

Supplementary Figure 4: Depletion of C-NHEJ proteins and measurement of the DSB removal rate

(a) Western blot analysis to estimate the level of 53BP1, Lig IV and PNKP in the NEs from HEK293 cells transfected with the specific or control siRNA. Lamin B was used as loading control in each case. qRT-PCR was performed using PNKP 3' UTR-specific oligos to confirm the depletion of endogenous PNKP by 3' UTR-specific siRNA. Normalization was performed with 18S, and data are represented as relative expression level (±SD) with the normalized value for control siRNA-treated samples considered as unity (n=2). **(b)** HEK293 cells were transfected with control siRNA and further mock- or Bleo- treated, kept for recovery for 0, 3, 6, 9 and 15 h after Bleo treatment, and harvested for genomic DNA isolation. Amplification of each large fragment (8-12 kb) was normalized to that of a small fragment (~200-400 bp) of the corresponding transcribed (HPRT1 and POLB) or non-transcribed (NANOG and OCT3/4) genes; the normalized data are represented (in the bar diagram) as the relative band intensity with the control siRNA/mock-treated sample arbitrarily set as 100 ($n \geq 3$; one representative gel figure is shown). Error bars represent ±SD of the mean.

Supplementary Figure 5

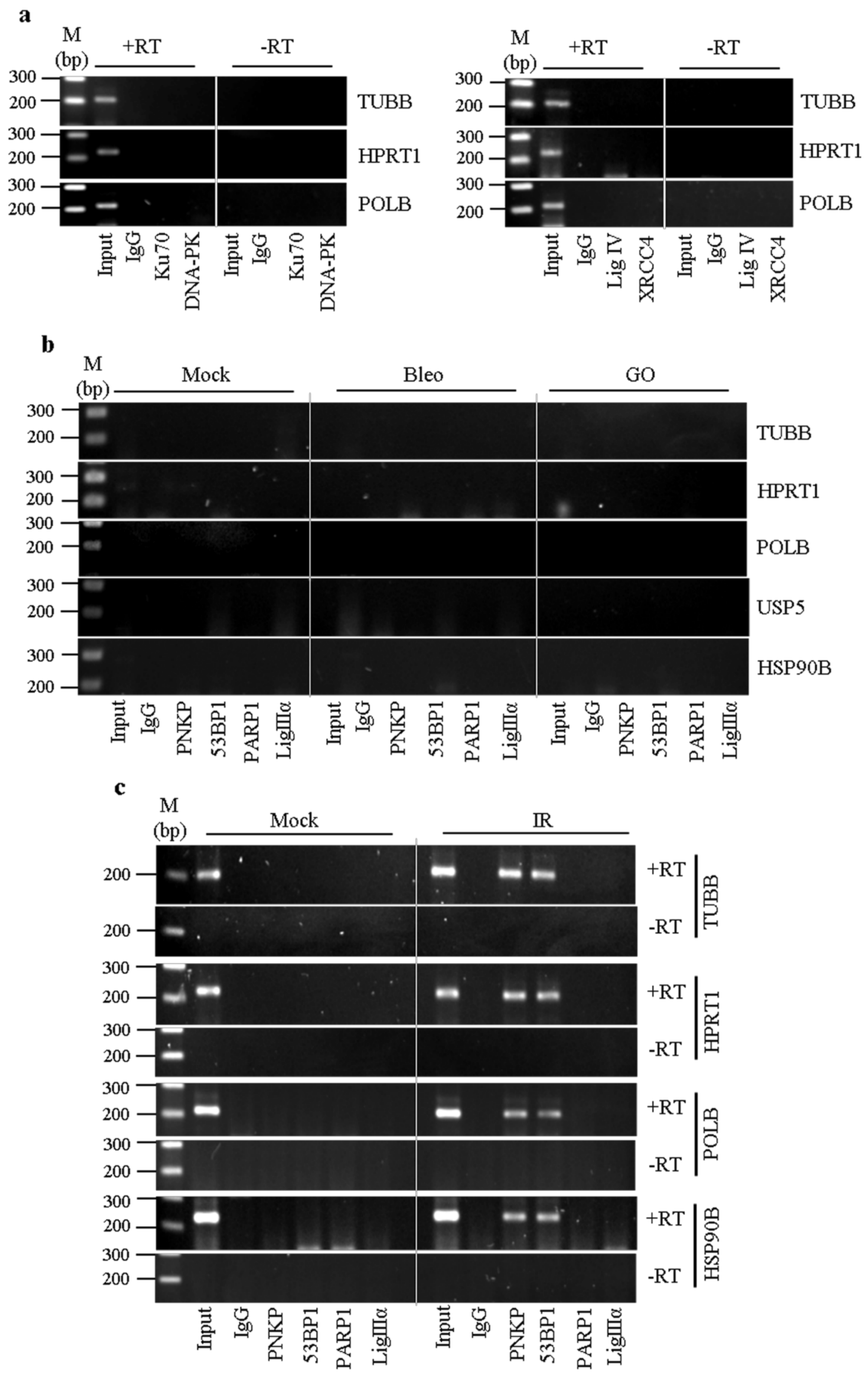

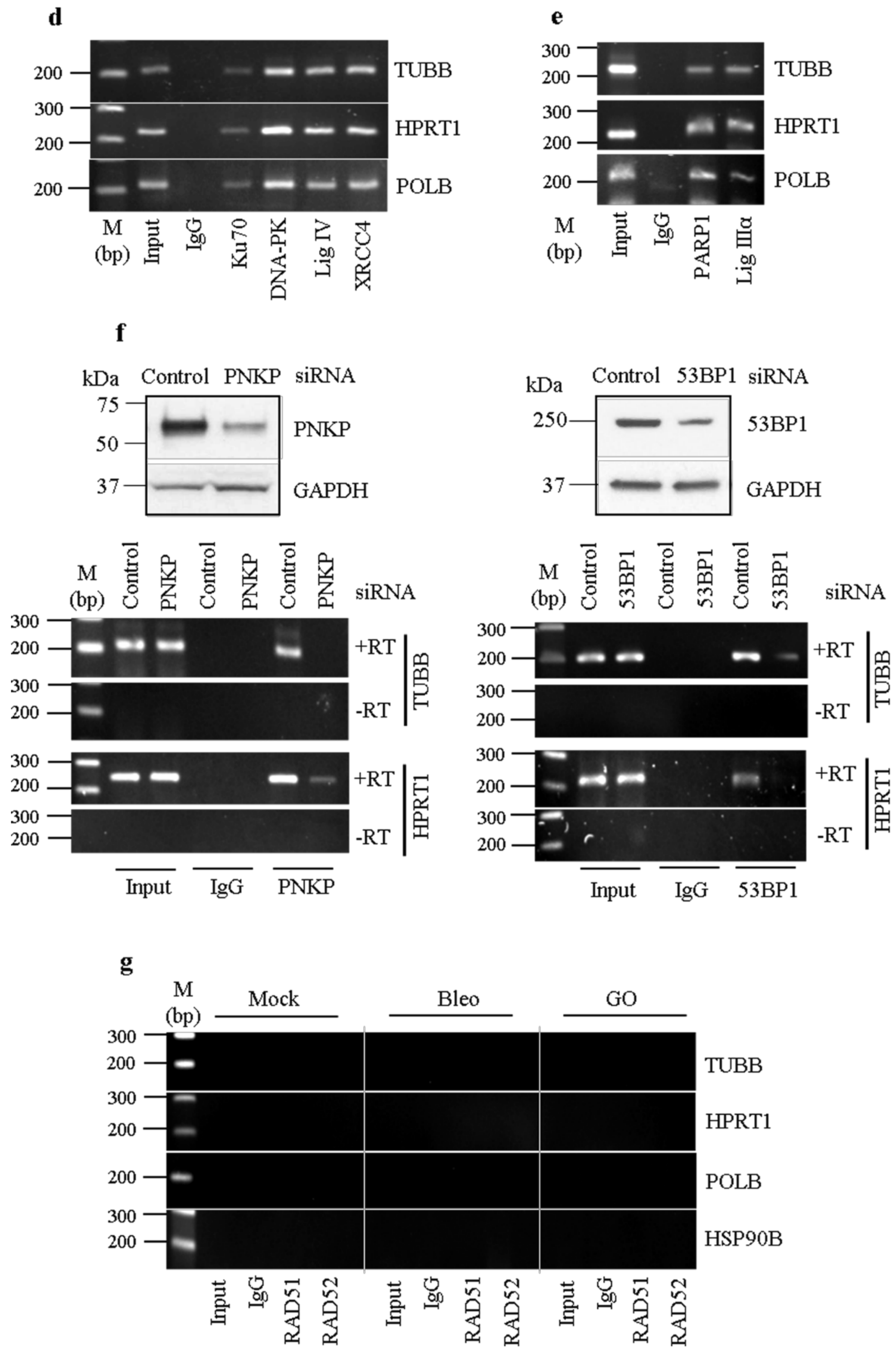
d
200
300
200
200
TUBB
HPRT1
POLB
M (bp)
Input
IgG
Ku70
DNA-PK
Lig IV
XRCC4
e
300
200
300
200
200
TUBB
HPRT1
POLB
M (bp)
Input
IgG
PARP1
Lig IIIα
f
kDa
Control
PNKP
siRNA
75
50
37
PNKP
GAPDH
kDa
Control
53BP1
siRNA
250
37
53BP1
GAPDH
M (bp)
Control
PNKP
Control
PNKP
Control
PNKP
siRNA
300
200
+RT
-RT
TUBB
+RT
-RT
HPRT1
Input
IgG
PNKP
M (bp)
Control
53BP1
Control
53BP1
Control
53BP1
siRNA
+RT
-RT
TUBB
+RT
-RT
HPRT1
Input
IgG
53BP1
g
M (bp)
Mock
Bleo
GO
300
200
TUBB
HPRT1
POLB
HSP90B
Input
IgG
RAD51
RAD52

h

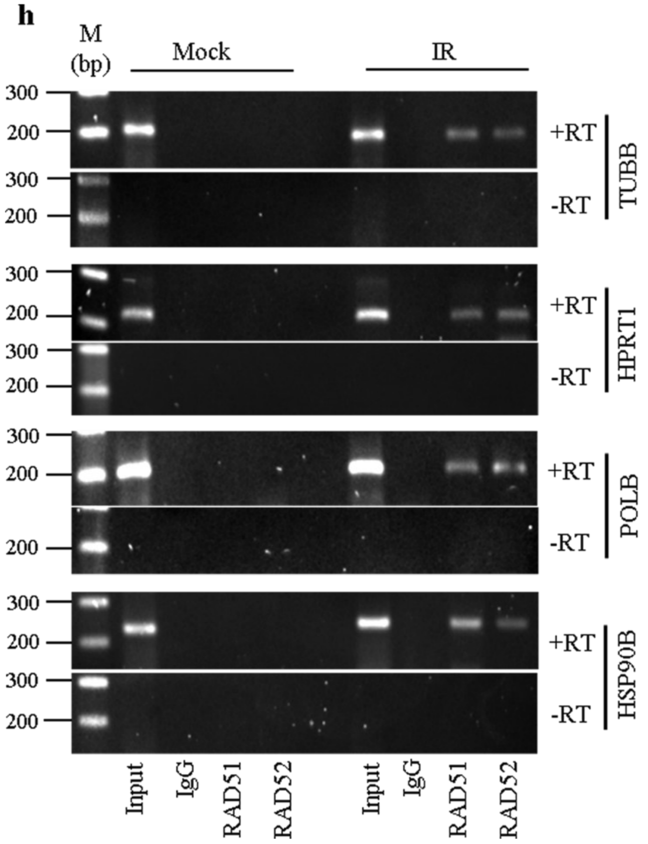

i

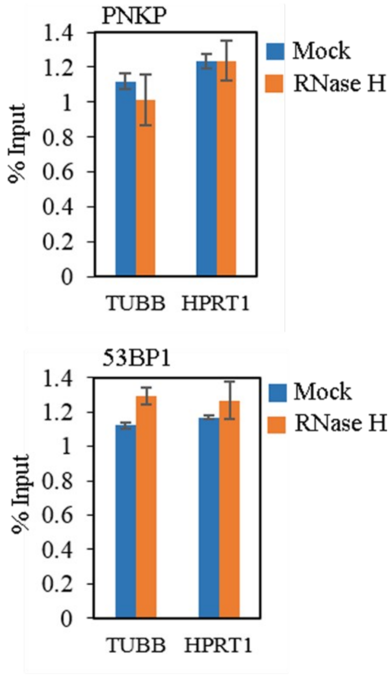

Supplementary Figure 5: Association of pre-mRNA with the C-NHEJ complex

(a) RNA-ChIP assays with Bleo-treated cells using Abs as indicated and analyzed by RT-PCR with intron-specific primers for TUBB, HPRT1 and POLB. **(b)** Control minus RT (-RT) reactions for RNA-ChIP assays performed with NE from mock/Bleo/GO-treated HEK293 cells, using the indicated Abs. RT-PCRs were carried out for TUBB, HPRT1, POLB, USP5 and HSP90B genes using intron-specific primers. **(c)** Similarly, HEK293 cells were exposed with mock/IR and subjected to RNA-ChIP assays, using the indicated Abs, and RT-PCRs were performed with +RT and -RT reactions for 4 genes. DNA-ChIP assays with Bleo-treated HEK293 cells using specific Abs against IgG, Ku70, DNA-PK, XRCC4 (**d**) and IgG, PARP1 and Lig IIIα (**e**) followed by PCRs using intron-specific primers for TUBB, HPRT1 and POLB. **(f)**, Upper panels, Western blot analysis to estimate the level of PNKP (left) and 53BP1 (right) in cell extracts from HEK293 cells transfected with control or specific target siRNAs. GAPDH was used as a loading control in

cell extracts from HEK293 cells transfected with control or specific target siRNAs. GAPDH was used as a loading control in each case. Lower panels, RNA-ChIP using anti-PNKP (left) and -53BP1 (right) Abs in respective control or depleted cells, followed by RT-PCR analysis using intron-specific primers for TUBB and HPRT1. **(g)** Control minus RT reactions for RNA-ChIP assays performed with NE from mock/Bleo/GO-treated cells, using anti-RAD51 and -RAD52 Abs. RT-PCRs were carried out for TUBB, HPRT1, POLB and HSP90B genes using intron-specific primers. **(h)** HEK293 cells were exposed to mock/IR and subjected to RNA-ChIP assay, using anti-RAD51 or -RAD52 Abs and RT-PCRs were performed for TUBB, HPRT1, POLB and HSP90B. **(i)** Bleo-treated HEK293 cells were mock- or RNase H-treated and subjected to DNA-ChIP assays using anti-PNKP or -53BP1 Abs. Real-time qPCRs were performed using intron-specific primers for TUBB and HPRT1. C_t values were used to calculate percent input and error bars represent ±SD of the mean. In each RNA-ChIP assay, 1% input was collected before IPs. All the experiments were repeated at least three times, and a representative image is shown; M represents a 1 kb DNA ladder.

Supplementary Figure 6

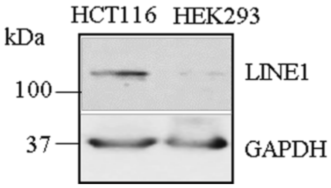

Supplementary Figure 6: Estimation of LINE1 (L1TD1)

Western blot to estimate the level of LINE1 (Ab: GTX32026, GeneTex) in the whole-cell extracts from HEK293 and HCT116 cells. GAPDH was used as a loading control.

Supplementary Figure 7

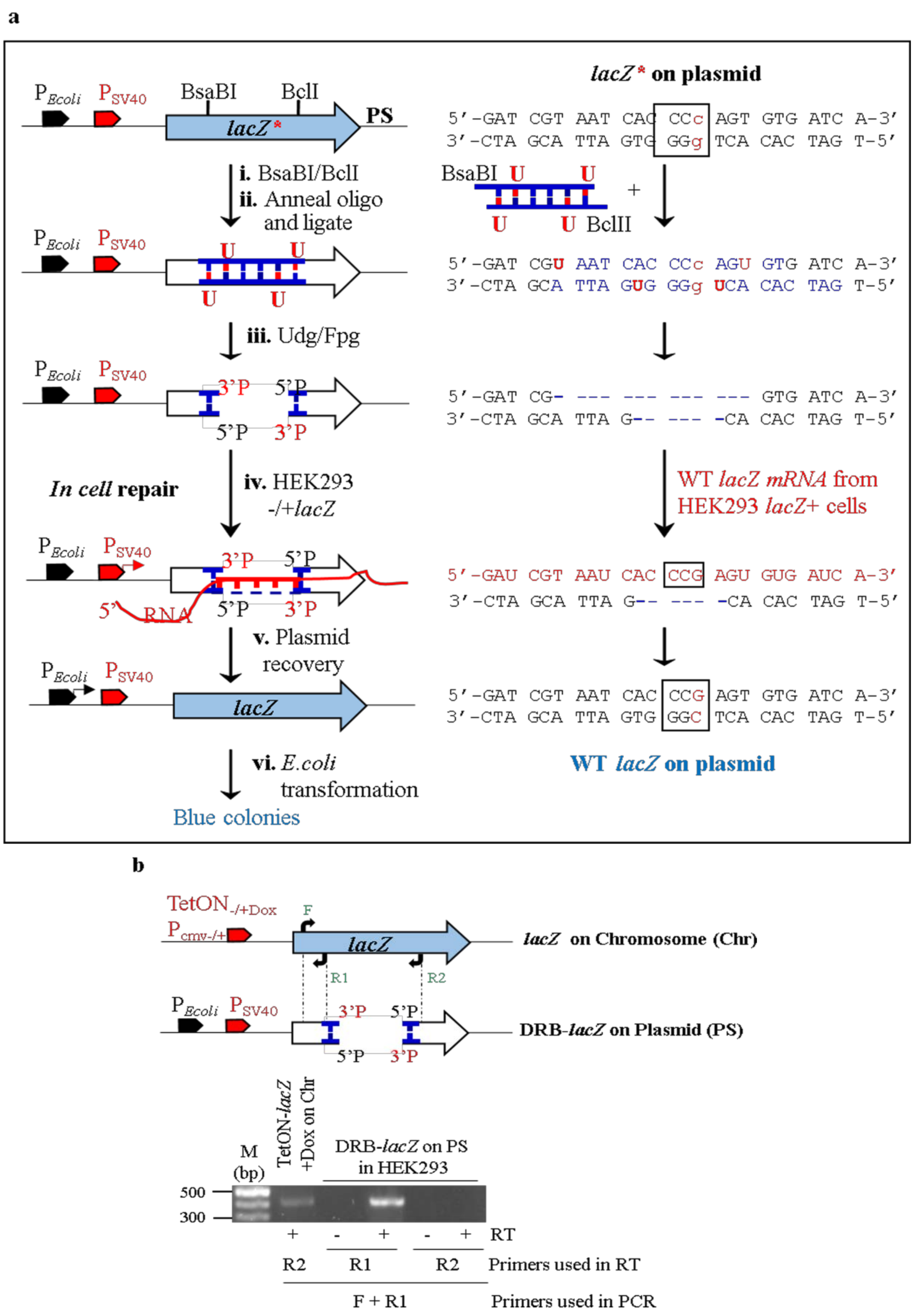

c

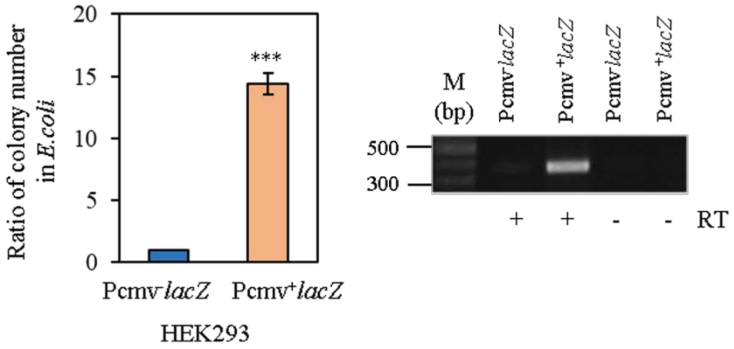

d

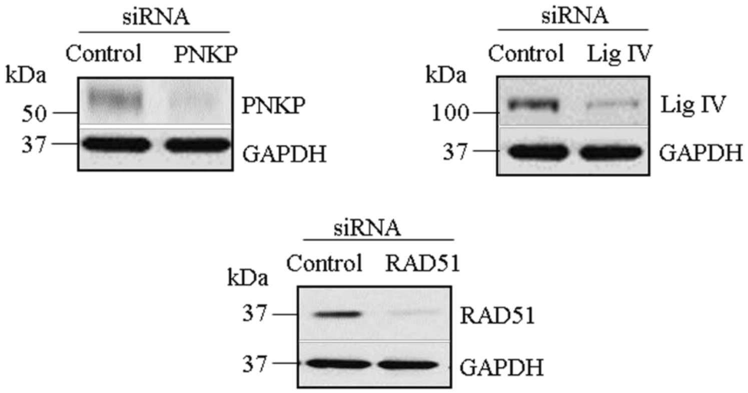

Supplementary Figure 7: Plasmid-based assay for RNA-templated DSBR

(a) Left panel, a uracil-containing duplex oligo (sequence in the right panel) was introduced within a *lacZ* gene (blue arrow) under the control of both *E. coli* and mammalian promoters using the unique *Bsa*BI/*Bcl*I sites. Plasmids were treated with *E. coli* Udg/Fpg to create DSBs with non-ligatable 3’-P termini[40], and several nucleotides were deleted in the process. Linearized plasmids were then transfected into HEK293 cells stably expressing *lacZ* (or corresponding control cells), and allowed to repair for 16h. Plasmids were then recovered from the transfected cells and introduced into *rec⁻lacZ⁻E. coli*; blue colonies were selected and the WT *lacZ* genomic sequence was confirmed by sequencing. Right panel, a silent mutation was introduced into the proline codon (CCG was replaced with CCC, marked in red) within the *lacZ* gene of the

transfected plasmid to distinguish it from the endogenous WT *lacZ*. This figure also shows how the gapped mutant plasmid is repaired using endogenous *lacZ* RNA as a template (in red) to generate WT *lacZ*. **(b)** RT-PCR showing the generation of truncated *lacZ* transcript from the DSB-containing plasmid. A cartoon on the top shows the location and direction of the primers (green) with black arrows within *lacZ*, either to amplify *lacZ* transcribed from chromosomal DNA of HEK293-TetON-lacZ+Dox cells (as a positive control) or the DSB-containing plasmid transfected in WT HEK293 cells. Primers R1 (TV286-R) and R2 (TV205-R) were used to synthesize cDNA from total RNA isolated either 12 h post-transfection of the DSB-containing plasmid or from Dox-treated control HEK293-TetON-lacZ+ cells. Primers R1 and F (TV209-F) were used in the subsequent PCR. The agarose gel picture with all the PCR products is shown at the bottom. The sequences of the primers are given in Supplementary Table 1. **(c)** Plasmid-based repair assay data are represented as the ratio of the number of *E.coli* blue colonies harboring plasmid DNA that was recovered after 16 h of repair in HEK293-Pcmv^{+}*lacZ* vs HEK293-Pcmv^{-} *lacZ* stable cell lines (after normalizing for the transfection efficiency and copy number variations of the *lacZ* gene in both cell lines). The number of *E. coli* blue colonies after transformation of the plasmids isolated from HEK293-Pcmv^{-}*lacZ* was arbitrarily set as 1. The data in the graph are the average of at least 3 independent experiments with estimated ***=P<0.005. RT-PCR, using primer TV205-R to synthesize cDNA from total RNA, showed the expression of *lacZ* in HEK293 (-/+Pcmv) *lacZ* cells (bottom panel). The PCR after RT was done using TV200-F and TV201-R. All primer sequences are given in Supplementary Table 1. **(d)** Western blots showing the levels of PNKP, Lig IV and RAD51 proteins in control vs specific siRNA-treated HEK293-Pcmv^{+}*lacZ* cells (see Methods). GAPDH protein was used as a loading control on the SDS-PAGE gel.

Supplementary Figure 8. Uncropped scans of the most important blots

Figure 1a

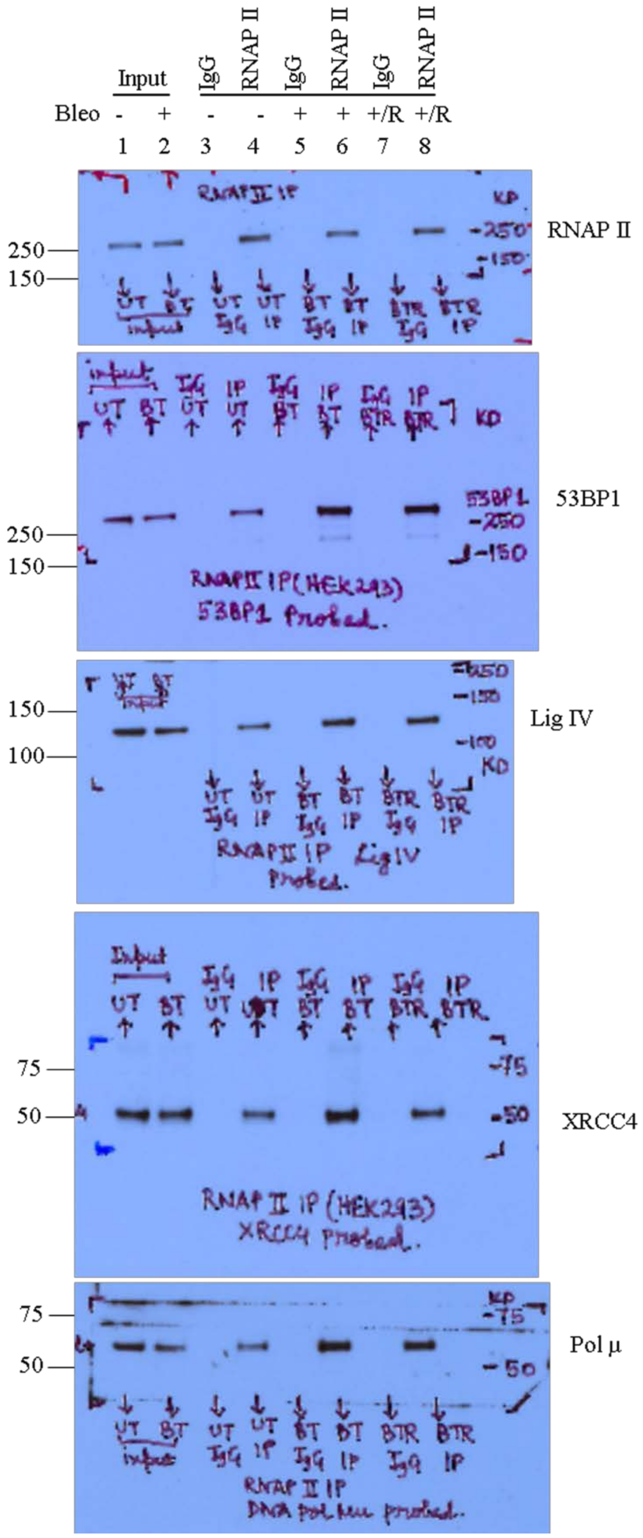

Figure 1a Cont.

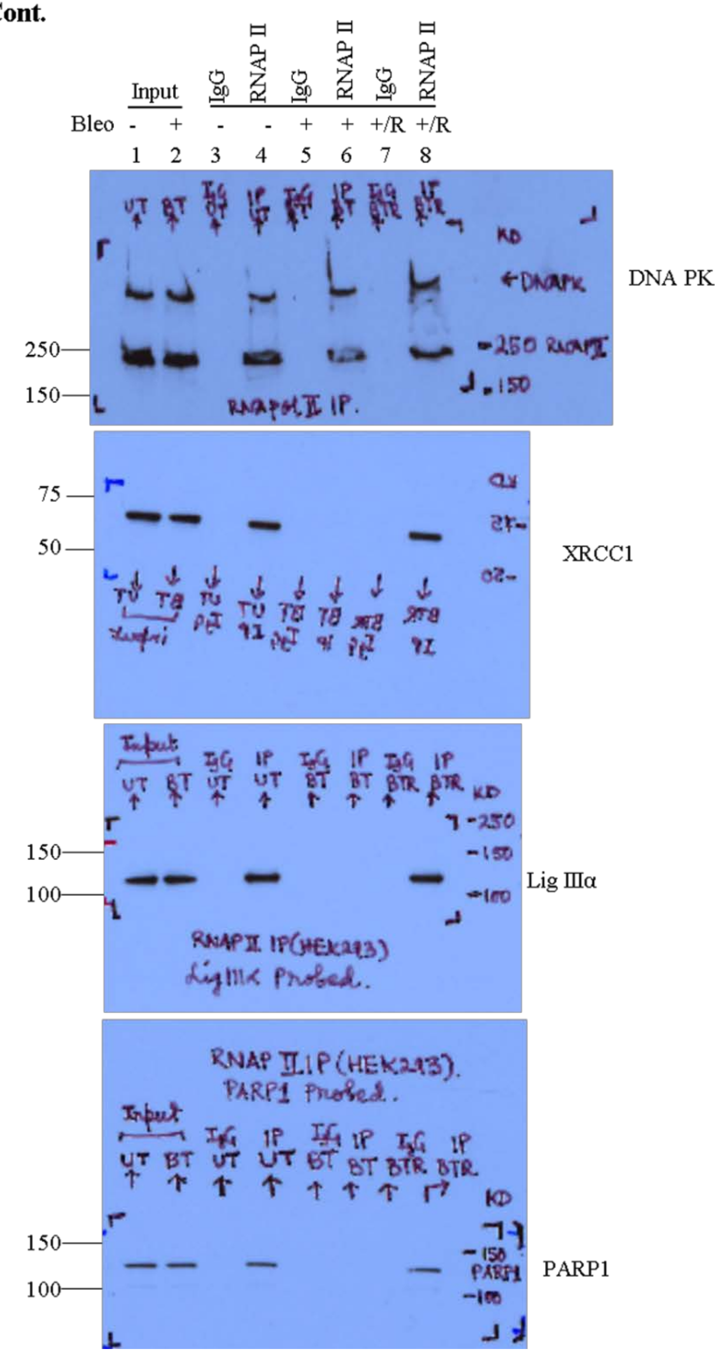

Figure 1b

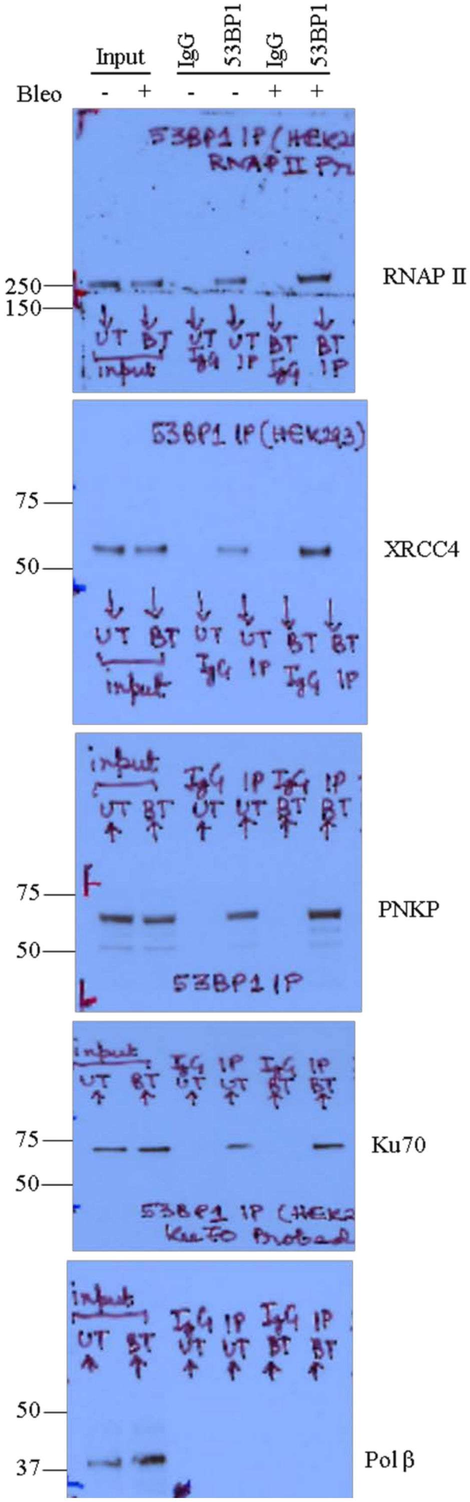

Figure 1c

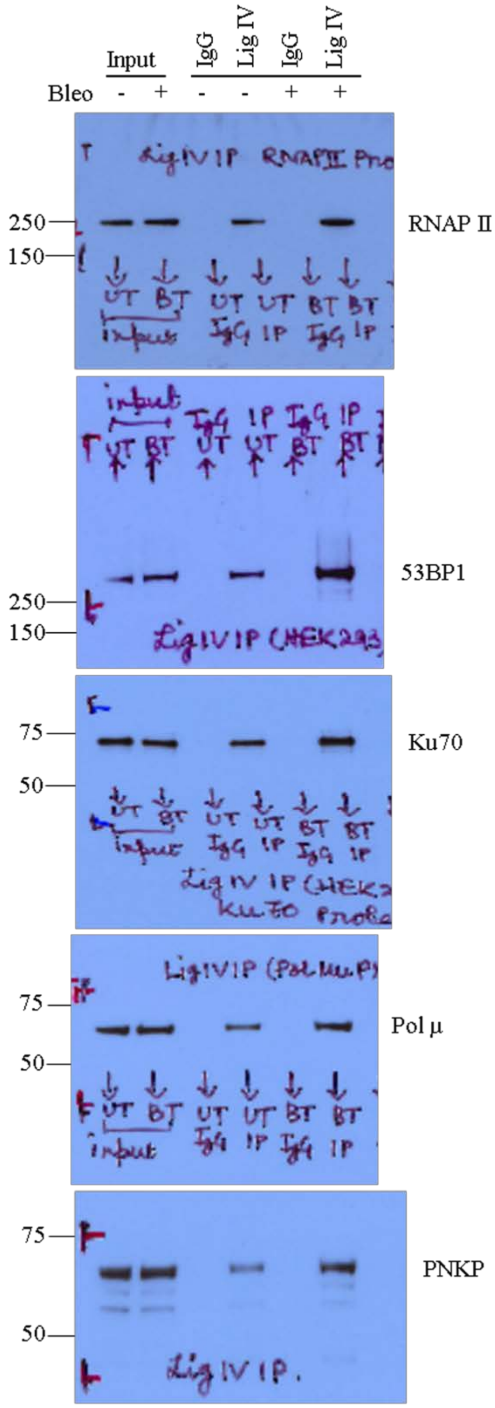

Figure 1d

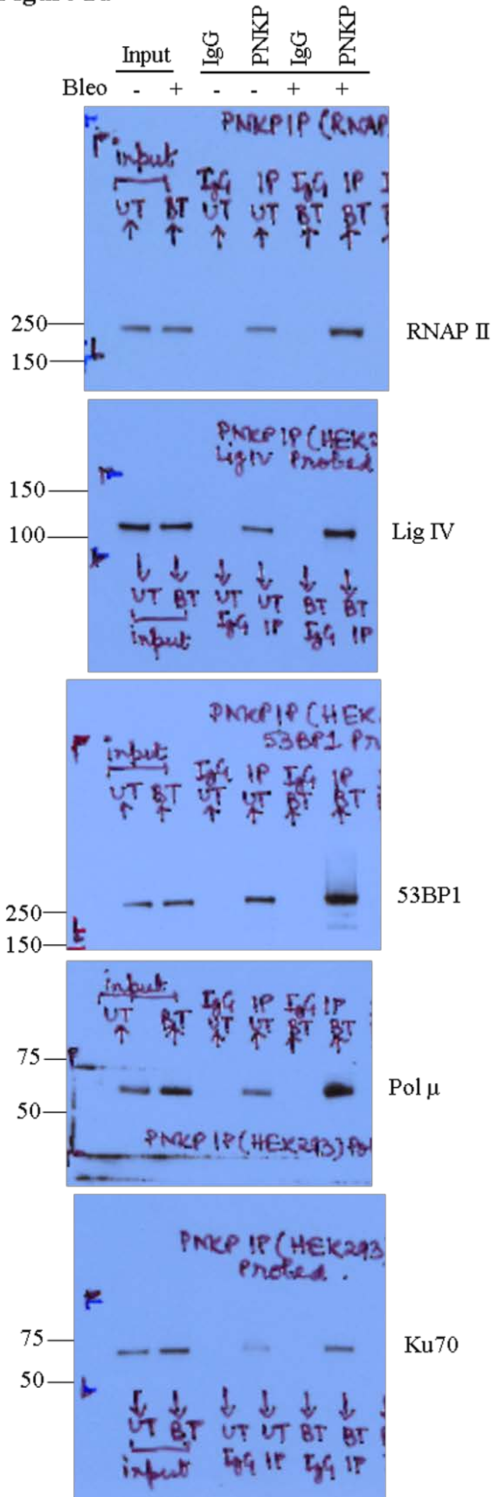

Figure 1e

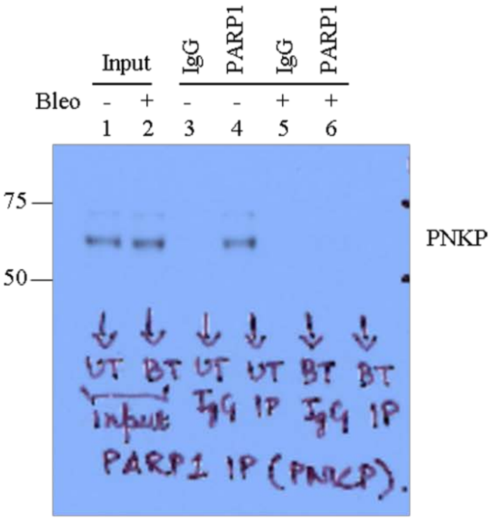

Figure 1f

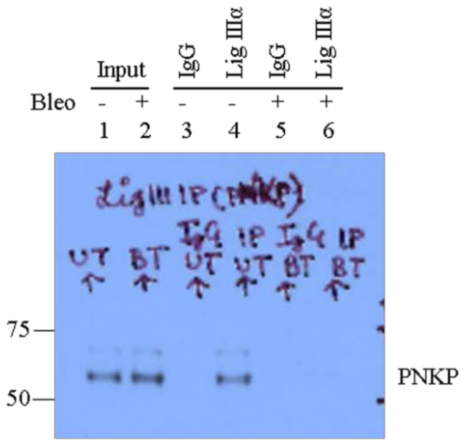

Supplementary Table 1: Primers used in the study

**Primers**	**Gene**	**Nucleotide sequence 5′ - 3′**	**Purpose**
TV001	n/a	GCTTAGCTTGGAATCGTATCATG TA(P)	RNA/DNA templated total repair (3’P-25 mer oligo)
TV031	n/a	GGCACGGTCTACACGGCACACG AGTGTACATGATACGATTCCAA GCTAAGC	DNA templated total repair (DNA 51 mer oligo)
TV56-F	MyoD	CCCCGACGGCTCTCTCTGCT	qPCR from gDNA (0.281 kb)
TV57-R	MyoD	GTGGTCTTGCGCTTGCACGC	qPCR from gDNA (0.281kb)
TV108-F	PNKP	CGGTCCAAGCTTGGCACCCAGG ATGGGCGAGGTGGAGGCCCC	Construction of pTV54 and pTV61
TV109-R	PNKP	AGAATAGGATCCGCCCTCGGAG AACTGGCAGTACA	Construction of pTV54
TV110-R	PNKP	AGAATATCTAGATTACTTGTCAT CGTCGTCCTTGTAGTC	Construction of pTV61
TV140-F	POLB	AGTGGGCTGGATGTAACCTG	SA-PCR (0.192 kb)
TV141-R	POLB	CCAGTAGATGTGCTGCCAGA	SA-PCR (0.192 kb)
TV142-F	HPRT1	TGGGATTACACGTGTGAACCAACC	LA-qPCR (10.4 kb)
TV143-R	HPRT1	GCTCTACCCTCTCCTCTACCGTC C	LA-qPCR (10.4 kb)
TV144-F	HPRT1	TGCTCGAGATGTGATGAAGG	SA-PCR (0.286 kb)
TV145-R	HPRT1	CTGCATTGTTTTGCCAGTGT	SA-PCR (0.286 kb)
TV161-F	POLB	CATGTCACCACTGGACTCTGCAC	LA-qPCR (12.2 kb)
TV162-R	POLB	CCTGGAGTAGGAACAAAAATTG CT	LA-qPCR (12.2 kb)
TV199-F	*E.coli lacZ*	GGCGACTTCCAGTTCAACATCA	Sequencing
TV200-F	*E.coli lacZ*	CATTATCCGAACCATCCGCTGTG	PCR, qPCR from gDNA and Sequencing
TV201-R	*E.coli lacZ*	CTGGTCTTCATCCACGCGCGCG	PCR, qPCR from gDNA and Sequencing
TV205-R	*E.coli lacZ*	CGCTATGACGGAACAGGTATT	RT-PCR
TV206-R	*E.coli lacZ*	CCGCATCAGCAAGTGTATCT	PCR from gDNA and Sequencing
TV209-F	*E.coli lacZ*	GTGCCGGCTAGCTGGCTGGAGT GCGATCTTCCT	Sequencing; RT-PCR
TV210-R	*E.coli lacZ*	TCCTGGGGATCCAGACATGATA AGATACATT	Construction of pTV108 and PCR from gDNA
TV213-F	*E.coli lacZ*	CGTAGCTAGCCAGGCTGGGACA CT	Construction of pTV108 and PCR from gDNA
TV214-F	NANOG	CTCCGGAATGGTAGTCTGAGAA GAA	LA-qPCR (8.6 kb)
TV215-R	NANOG	ATTTAGGGCAGGCACAAGATGG	LA-qPCR (8.6 kb)
TV218-F	HSP70	GTCAACATGGTGAAATCCCGTC TCTACTA	LA-qPCR (8.9 kb)

TV219-R	HSP70	TAGGAAATGCAAAGTCTTGAAG CTCCAAA	LA-qPCR (8.9 kb)
TV232-F	OCT3/4	TCTGTGGCCTCACCCTATGA	LA-qPCR (10.1 kb)
TV233-R	OCT3/4	CAGACCTGTGGCAGGTATTGAA	LA-qPCR (10.1 kb)
TV247-F	*E.coli lacZ*	CAGGCTGGGAGCGGCCGCATGA GCGAAAAATACATCG	Construction of pTV119
TV248-R	*E.coli lacZ*	CCTGGATCCTTATTATTATTTTT GACACCAGACCAACT	Construction of pTV119
TV263-F	*E.coli lacZ*	(P)UAATCACCC**c**AGUGT	DSBR plasmid assay
TV264-R	*E.coli lacZ*	(P)GATCACACUGGGGUGATTA	DSBR plasmid assay
TV265-F	*E.coli lacZ*	(P)TAATCACCCCAGTGT	Construction of pTV123
TV266-R	*E.coli lacZ*	(P)GATCACACTGGGGTGATTA	Construction of pTV123
TV267	n/a	(P)CGTGTGCCGTGTAGACCGTGC C	RNA/DNA templated total repair (5'P-22 mer oligo)
TV286-R	*E.coli lacZ*	GCCACATATCCTGATCTTCCAGA T	Sequencing; RT-PCR
TV287-F	*E.coli lacZ*	CTCTGGATGTCGCTCCACCAGGT AAACAG	Sequencing
TV07 RPmix	NANOG	RealTimePrimers.com	SA-PCR (0.244 kb), ChIP qPCR and Checking transcription in HEK293
TV08 RPmix	HSP70	RealTimePrimers.com	SA-PCR (0.203 kb)
TV10 RPmix	OCT3/4	RealTimePrimers.com	SA-PCR (0.455 kb) and Checking transcription in HEK293
TV17 RPmix	POLB	RealTimePrimers.com	Checking transcription in HEK293
TV19 RPmix	GAPDH	RealTimePrimers.com	Checking transcription in HEK293; ChIP qPCR
TV21 RPmix	NeuroD	RealTimePrimers.com	Checking transcription in HEK293 and qChIP Assay
TV20 RPmix	β-Actin	RealTimePrimers.com	ChIP qPCR
RNA oligo 51 Mer	n/a	**GGCA**rCrGrGrUrCrUrArCrArCrGrG rCrArCrArCrGrArGrUrGrUrArCrAr UrGrArUrArCrGrArUrUrCrCrArArG rCrU**AAGC**	RNA templated total repair (51 mer oligo) (DNA bases are in bold)
TUBB-In2-For	TUBB	TCCCTGTCTCCCACTTATCTG	(RNA/DNA)-ChIP PCR/qPCR
TUBB-In2-Rev	TUBB	TCTGGCAGAAGGGAAGGTTT	(RNA/DNA)-ChIP PCR/qPCR
HPRT1-In2-For	HPRT1	TGCCAGTATGGGTGGGAGAA	(RNA/DNA)-ChIP PCR/qPCR
HPRT1-In2-Rev	HPRT1	AGGGTAAAAACCCAGGCATGA	(RNA/DNA)-

			ChIPPCR/qPCR
PolB-int1-For	POLB	CCTGCCCTTAGCCCTCTTTT	(RNA/DNA)-ChIP PCR/qPCR
PolB-int1-Rev	POLB	AAGGAGGGAGCACAATCAGC	(RNA/DNA)-ChIP PCR/qPCR
USP5-In2-For	USP5	CCCTTTCCTACCAAGCCTGT	RNA-ChIP PCR/qPCR
USP5-In2-Rev	USP5	CCCTTGCACAGAAGGGACTT	RNA-ChIP PCR/qPCR
HSP90B-In2-For	HSP90B	ACAGAATGTGCCCACAGCTT	RNA-ChIP PCR/qPCR
HSP90B-In2-Rev	HSP90B	CGAAGGCAGGGGAAGGTAAA	RNA-ChIP PCR/qPCR
PNKP3UTR FP	PNKP (endogenous)	GAGATCCCGTTCCGGCTATG	PNKP 3UTR specific qRT-PCR
PNKP3UTR RP	PNKP (endogenous)	CAGCGTTTATTGTGGAGGGG	PNKP 3’UTR specific qRT-PCR
HSP 70 FP	HSP70	CTGTGCGGCTGCAGGCACCGGC	DNA ChIP qPCR
HSP 70 RP	HSP70	TGGTGCGGTTGCCCTGGTCGTT	DNA ChIP qPCR
HSP89 FP	HSP89	CCTCTGTAGACGTCCTGCAAGGT	DNA ChIP qPCR
HSP89 RP	HSP89	ATCCGATTCTGGGTTAATAAGTG	DNA ChIP qPCR
18S Primer mix	18S	RealTimePrimers.com	Endogenous control in qRT-PCR.

Supplementary Table 2: Number of blue colonies obtained in all *lacZ* plasmid-based assays

Mammalian cells from which the repaired *lacZ*-containing plasmid was isolated		Number of blue colonies in *E.coli**
none**		0
HEK293		0
HEK293-TetON*lacZ*	- Dox	5 ±0.6
	+ Dox	144 ±6.5
HEK293-*lacZ*	$Pcmv^-$	8 ±1
	$Pcmv^+$	155 ±10.4
HEK293-$Pcmv^+$*lacZ*		
	Control siRNA	241 ±13
	PNKP siRNA	9 ±2.5
	Lig IV siRNA	23 ±5
	RAD51 siRNA	218 ±9.8

* Results represent the average number of colonies (± SD) obtained with plasmid DNA recovered from 3 independent mammalian cell transfections of the DSB-containing plasmid.

** Udg/Fpg digested DSB-containing plasmid was tested for colony formation in *E.coli* prior to transfection into mammalian cells.
